# Analysis of sugar components and identification of *SPS* genes in citrus fruit development

**DOI:** 10.3389/fpls.2024.1372809

**Published:** 2024-03-28

**Authors:** Wen Lu, Wenhui Hao, Kexin Liu, Jiahuan Liu, Chunmei Yin, Yujiao Su, Zhiyu Hang, Bin Peng, Huan Liu, Bo Xiong, Ling Liao, Jiaxian He, Mingfei Zhang, Xun Wang, Zhihui Wang

**Affiliations:** ^1^ College of Horticulture, Sichuan Agricultural University, Chengdu, Sichuan, China; ^2^ College of Agricultural, Sichuan Nationalities University, Liangshan Yi autonomous prefecture, Sichuan, China

**Keywords:** citrus, sugar metabolism, sucrose phosphate synthase, fruit quality, expression network

## Abstract

Sugar is a primary determinant of citrus fruit flavour, but undergoes varied accumulation processes across different citrus varieties owing to high genetic variability. Sucrose phosphate synthase (SPS), a key enzyme in glucose metabolism, plays a crucial role in this context. Despite its significance, there is limited research on sugar component quality and the expression and regulatory prediction of *SPS* genes during citrus fruit development. Therefore, we analysed the sugar quality formation process in ‘Kiyomi’ and ‘Succosa’, two citrus varieties, and performed a comprehensive genome-wide analysis of citrus *CsSPSs*. We observed that the accumulation of sugar components significantly differs between the two varieties, with the identification of four *CsSPSs* in citrus. *CsSPS* sequences were highly conserved, featuring typical SPS protein domains. Expression analysis revealed a positive correlation between *CsSPS* expression and sugar accumulation in citrus fruits. However, *CsSPS* expression displays specificity to different citrus tissues and varieties. Transcriptome co-expression network analysis suggests the involvement of multiple transcription factors in shaping citrus fruit sugar quality through the regulation of *CsSPSs*. Notably, the expression levels of four *CsWRKYs* (*CsWRKY2*, *CsWRKY20*, *CsWRKY28*, *CsWRKY32*), were significantly positively correlated with *CsSPSs* and *CsWRKY20* might can activate sugar accumulation in citrus fruit through *CsSPS2*. Collectively, we further emphasize the potential importance of *CsWRKYs* in citrus sugar metabolism, our findings serve as a reference for understanding sugar component formation and predicting *CsSPS* expression and regulation during citrus fruit development.

## Introduction

Citrus, a major global fruit crop, is rich in nutrients such as sugars, organic acids, amino acids, carotenoids, and flavonoids (Guo et al., 2016; Sheng et al., 2017). Sugar metabolism profoundly impacts citrus fruit quality, with sugar content generally increasing during fruit development (Bush, 2020). ‘Kiyomi’ (*Citrus unshiu × sinensis*) and ‘Succosa’ (*Citrus reticulata Blanco cv. Succosa*) are important citrus cultivars in Sichuan Province, due to their good fresh-eating quality. However, the sweetness of Kiyomi’s fruit varies greatly before and after ripening, whereas Succosa is stable, relatively. Usually, the sugar amounts in developing fruits significantly vary among citrus cultivars owing to their high genetic variability (Albertini et al., 2006).

Soluble sugar, which is composed of sucrose, fructose, and glucose, constitutes the main sugar in citrus fruits, with sucrose being the principal storage form (Ruan, 2014). Sucrose phosphate synthase (SPS) catalyses the conversion of fructose-6-phosphate (F-6-P) and UDP-glucose (UDP-G) to sucrose 6-phosphate (S-6-P), serving as a rate-limiting enzyme for sucrose synthesis (Winter and Huber, 2000; Coleman et al., 2009). Consequently, SPS plays a crucial role in controlling sucrose synthesis in leaf tissues and sucrose accumulation in fruits (Lunn, 2003).

In recent years, genome-wide data have helped identify *SPS* genes in plants, forming families with relatively few members. For example, *Arabidopsis thaliana* has four members, rice (*Oryza sativa*) has five, and pears have eight (Castleden et al., 2004; Okamura et al., 2011; Koramutla et al., 2019). Despite different *SPS* numbers in plants, their protein sequences are similar, containing conserved domains for sucrose synthesis, sugar transport-1, and S6PP. Phylogenetic analysis categorises *SPS* genes into four families (A, B, C, and D), with the D family exclusive to certain monocotyledonous plants (Langenkämper et al., 2002). Tissue-specific expression is observed, with *SPS1* and *SPS2* preferentially expressed in fruits, whereas *SPS3* and *SPS4* are expressed in leaves and flowers (Okamura et al., 2011). *SPS* expression directly influences plant sugar metabolism, with *SPS* overexpression impacting starch and sucrose proportions in transgenic tomato and *Arabidopsis* leaves (Worrell et al., 1991; Signora et al., 1998; Anur et al., 2020). Moreover, the *SPS* expression levels further affect plant morphogenesis by regulating glucose metabolism. For example, overexpression of the spinach *SPS* gene in cotton can improve cotton fibre quality, and overexpression of *SoSPS1* increases plant height and stem number of some transgenic sugarcane strains (Park et al., 2008; Anur et al., 2020).

In addition to factors such as low temperatures, drought, and hormones, transcription factors also regulate *SPS* expression (Reimholz et al., 2008; Roy Choudhury et al., 2008). For example, the ABA-associated *FaMRLK47* regulates sucrose and starch metabolism in strawberry fruit, and silencing the MYB transcription factor *FaGAMYB* decreases transcription levels of *FaSPS3* (Jia et al., 2017). Moreover, *FaMYB44.2* can regulate sucrose accumulation by inhibiting the expression of *FaSPS3* (Wei et al., 2018).

In this study, we comprehensively investigated the sugar and acid qualities of two citrus varieties (‘Kiyomi’ and ‘Succosa’) and analysed *CsSPSs* in the citrus genome. Through *CsSPSs* expression and co-expression analyses, we sought to elucidate their crucial role in sugar metabolism during citrus fruit development, as well as obtain valuable insights into the transcriptional regulation of *CsSPSs* and advance our understanding of citrus sugar metabolism pathways.

## Materials and methods

### Plant materials

Mandarin fruits (‘Kiyomi’ and ‘Succosa’) were harvested from a commercial orchard in Liangshan Yi autonomous prefecture, Sichuan province, China, ensuring uniform size and absence of visible injuries. Fruits harvested in September (Sep), October (Oct), November (Nov), and December (Dec)were transported to the laboratory, where pulp samples were frozen, homogenised in liquid nitrogen, and stored at −80°C for subsequent analyses. Three replicates, each consisting of six fruits, were analysed.

### Total soluble solid, titratable acidity

‘Kiyomi’ and ‘Succosa’ fruits at each stage were selected to determine the total soluble solid (TSS) and titratable acidity (TA). At each development stage, more than 18 fruits were used for quality assessment, with three replicates. TSS (%) and TA (%) were measured using a digital acidity metre (Pocket PAL-BXIACID1, ATAGO, Tokyo, Japan) following the manufacturer’s instructions.

### Determination of glucose, fructose, and sucrose contents

Glucose, fructose, and sucrose levels were determined, as described earlier (Liu et al., 2019). Briefly, 2 g of pulp was homogenised with 10 mL of ddH_2_O, incubated for 15 min at 80°C, centrifuged, and filtered. The resulting supernatant was analysed on an Agilent 1260 HPLC system (Agilent Technologies) with a refractive index detector using an Innoval NH_2_ column (4.6 mm × 250 mm, 5 μm, Agela Technologies, Shanghai, China). The mobile phase comprised acetonitrile: water (80:20, v/v) with a flow rate of 1 mL min^−1^.

### Identification and characteristic analysis of the *SPS* gene family

Citrus SPS genes were identified following established methods (Hu et al., 2015; He et al., 2016). Genomic sequences from various citrus species (*Citrus clementina* v1.0, *Citrus grandis* ‘Wanbaiyou’ v1.0, *Poncirus trifoliata* v1.0, and *Citrus sinensis* v2.0) were obtained from the Citrus Pan-genome to Breeding Database (CPBD: http://citrus.hzau.edu.cn/index.php). *Arabidopsis SPS* gene members and their protein sequences were sourced from the *Arabidopsis* Information Resource (TAIR: https://www.arabidopsis.org/browse/genefamily/index.jsp). HMMER software version 3.0 was utilised to identify *C. sinensis SPS* (*CsSPS*) genes. Furthermore, the MapGene2Chrom software (MG2C_v2.1) was employed to generate a chromosome location image of *CsSPSs*, and TBtools software was used to display the exon/intron structure of all *CsSPSs.*

### Analysis of gene structure and conserved motifs of *CsSPSs*

Conserved motifs of SPS proteins were identified using the multiple EM for motif elicitation (MEME software) (Brown et al., 2013). The optimal width of each motif ranged from 6–20, with a maximum of six motifs to search and default values for other parameter settings (Bailey et al., 2006). To ensure the inclusion of SPS domains, all candidate SPSs were validated using the National Center for Biotechnology Information (NCBI) Conserved Domain Database (CCD) to ensure that they contained the SPS domains. Additionally, the S6PP domain was predicted through multiple sequence alignment using BioEdit.

### Phylogenetic relationship analysis of the *CsSPS* gene family

Multiple sequence alignments of citrus and *Arabidopsis thaliana* SPS protein sequences were performed using Molecular Evolutionary Genetics Analysis (MEGA) version 6.0, with 1,000 bootstrap replications, pairwise deletion, and Poisson model. Subsequently, neighbour-joining phylogenetic trees were constructed.

### RNA-Seq data and qRT-PCR analysis

RNA-Seq data were retrieved from published studies (Terol et al., 2019; Feng et al., 2021; Zhang et al., 2021). The data that support the findings of this study have been deposited in the NCBI BioProject database under accession numbers PRJNA636131, PRJNA517400 and PRJEB12880. RNA-Seq data analysis was performed as described previously (Pertea et al., 2016). Briefly, the analysis included preprocessing for quality using FastQC, trimming low-quality reads (q < 20) and adapters using Trimmomatic, alignment to the *C. sinensis* genome using HISAT2 with default parameters, and assembly of mapped reads using StringTie. Fragments per kilobase per million mapped fragments (FPKM) was used to represent the gene expression levels. *CsSPS* expression profiles were extracted from the RNA-Seq data.

Total RNA was isolated from different tissues, as described previously (Liu et al., 2022). Specific primer pairs for *CsSPSs* amplification were designed using the Primer Express software (Applied Biosystems, Foster City, CA, USA). The specificity and amplification efficiency of the primers were validated using BLASTN against the sweet orange genome. Relative gene expression values, with the citrus β-actin gene as the internal reference gene, were calculated using the 2^−ΔΔCt^ method (Livak and Schmittgen, 2001). The sequences of RT-PCR primers are displayed in **Supplementary Table S5**. All expression data were processed using the Z-score standardisation method.

### Co-expression network analysis

Weighted gene co-expression network analysis (WGCNA) (v1.71) in R was used to construct the co-expression networks (Langfelder and Horvath, 2008). Among 29,138 genes, 16,961 with a sum FPKM < 1 across all samples were removed. The remaining genes were used for the WGCNA. The one-step network construction and module detection function were conducted using an unsigned topological overlap matrix (TOM), a soft-thresholding power b of 14 (R2 > 0.9), a minimal module size of 30, and a branch merge cut height of 0.25. The co-expression network of candidate *CsSPSs* was visualised using Cytoscape (version 3.6.1) (Shannon et al., 2003).

### Dual-luciferase activity assay

The WRKY response cis-acting elements (W-box) in the promoter regions (2000-bp upstream of the initiation codon) of *CsSPS1*, *CsSPS2*, *CsSPS3* and *CsSPS4* using PlantCARE online software (https://bioinformatics.psb.ugent.be/webtools/plantcare/html/) were estimated (**Supplementary Table S4**). Afterwards, *CsSPS2* promoter were inserted independently into the pGreen II 0800-LUC double-reporter vector, and the CDSs of *CsWRKY2*, *CsWRKY20*, *CsWRKY28* and *CsWRKY32* were inserted into the constructed pGreen 62-SK vector driven by the 35S promoter as the effector, using the primer sequences listed in **Supplementary Table S7**. The constructed effector and each reporter plasmid were co-transformed into tobacco leaves using A. tumefaciens strain GV3101(psoup-p19). Plasmids containing *CsWRKYs* and promoter were combined at a 10:1 ratio (v/v) and then infiltrated into tobacco leaves using needleless syringes. At 2 days after infiltration at 21 ^◦^C, the LUC and REN activities were measured using a Dual-Luciferase Assay kit (Promega, USA) on a Luminoskan Ascent Microplate Luminometer (Thermo, USA). The results were calculated using the LUC to REN ratio. Six biological repeats were assayed for each combination.

### Statistical analysis

All data are presented as the mean (± standard deviation [SD]) of a representative experiment. Significant differences between samples were determined using ANOVA followed by Tukey’s test. The heatmaps were plotted using R studio software using the pheatmap package. The correlation analysis was performed using R studio software. Figures were created using GraphPad Prism (GraphPad Software, CA, USA).

## Results

### Sugar and organic acid contents in citrus fruit

**Figure 1:**
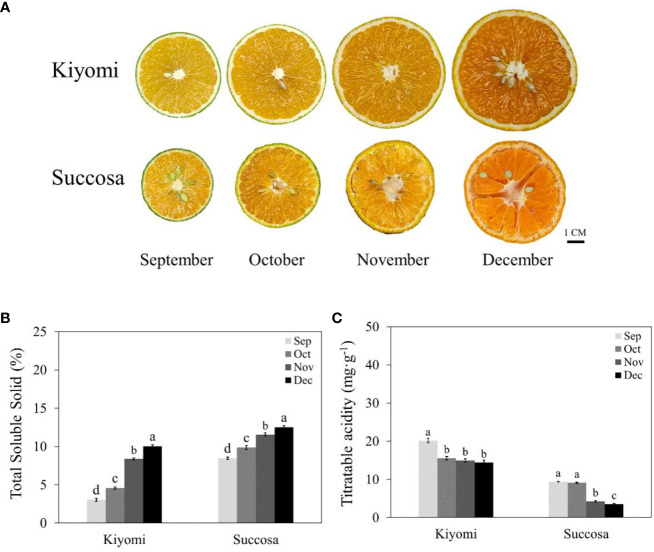
Citrus fruit phenotype. **(A)** Developmental stages of citrus fruit (‘Kiyomi’ and ‘Succosa’). **(B)** TSS. **(C)** TA. TSS, total soluble solid; TA, titratable acid. The different letters represent the significant differences between three groups during storage (P <0.05).

To comprehend the dynamics of sugar and acid development in citrus, we assessed TSS and TA in two citrus varieties (‘Kiyomi’ and ‘Succosa’). As citrus fruit matured, the pulp colour deepened (**Figure 1A**), TSS content exhibited an evident upward trajectory (**Figure 1B**), and TA content significantly decreased (**Figure 1C**). Notably, the accumulation stages of sugar and acid varied among different citrus varieties. ‘Succosa’ attained higher TSS levels and lower TA content earlier than ‘Kiyomi’, which maintained stability. In contrast, ‘Kiyomi’ demonstrated a faster TSS accumulation rate and significantly higher TA content compared with that of ‘Succosa,’ despite lower TSS levels.

### Sucrose, glucose, and fructose contents

**Figure 2:**
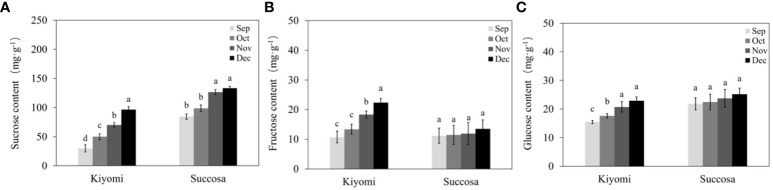
Soluble sugar component in the pulp of citrus fruit. **(A)** Sucrose. **(B)** Fructose. **(C)** Glucose. The different letters represent the significant differences between three groups during storage (P <0.05).

As the primary sugars in citrus fruits, the formation of soluble sugars, including sucrose, fructose, and glucose, were analysed for quality assessment during development (**Figure 2**). We identified sucrose as the predominant component of soluble sugar in citrus pulp, followed by glucose and fructose. All three sugar components increased with citrus fruit development, with sucrose exhibiting the most substantial increase. ‘Succosa’ displayed higher sucrose content and consistent glucose and fructose levels compared with that of ‘Kiyomi.’ However, ‘Kiyomi’ exhibited a faster accumulation rate of sucrose, glucose, and fructose, aligning with the TSS results.

### Identification of citrus *SPS* genes

**Figure 3:**
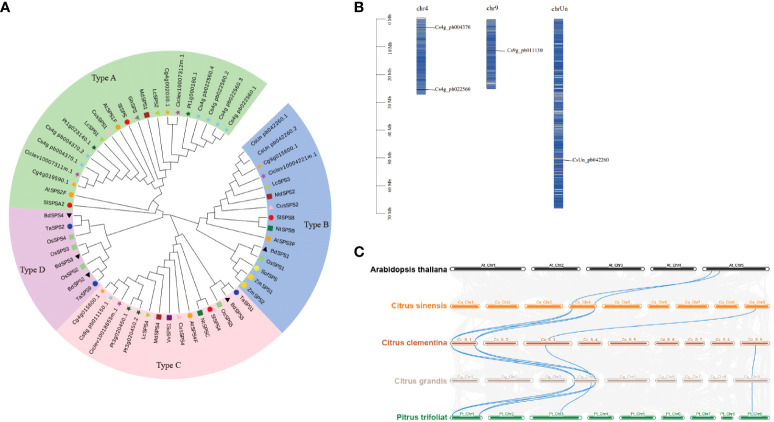
Phylogenetic and genomic analysis of SPS proteins. **(A)** Phylogenetic relationship of SPS proteins among Citrus and other species. **(B)** Chromosome distribution of *CsSPSs*. **(C)** Collinearity analysis of *SPS* genes from *Arabidopsis* and Citrus. Phylogenetic analysis was performed using the neighbour-joining method in MEGA v5.1. SPS proteins in *C. sinensis, C. clementina, C. grandis,* and *P. trifoliata* are represented using skyblue star, yellow circles, magenta star, orange star, and dark green star, respectively. Different species and type using different shape and color.

Sucrose, a vital sugar in citrus fruits, is synthesised by SPS, the rate-limiting enzyme. We analysed the *CsSPS* family from four representative citrus varieties (*Citrus sinensis, Citrus clementina, Citrus grandis, and Pitrus trifoliat*) (**Supplementary Table S1**) and constructed phylogenetic trees to elucidate the evolutionary relationship of *SPS* genes between citrus and other species (**Supplementary Table S2**). Citrus SPS genes were categorised into three families, with *CsSPS1* (Cs4g_pb004370) and *CsSPS2* (Cs4g_pb022560) in family A, *CsSPS3* (CsUn_pb042260) in family B, and *CsSPS4* (Cs9g_pb011150) in family C (**Figure 3A**). *CsSPS3* demonstrated a closer relationship with *Arabidopsis AtSPS3F* within the B family, while *CsSPS4* also exhibited a closer relationship with *Arabidopsis AtSPS4F* within the C family. The chromosomal distribution of *CsSPSs* was analysed based on the physical location data retrieved from the GCA_018104345.1_Cs2.0_genomic database on the NCBI website. Using Dual Synteny Plotter software, we assessed the syntenic relationship between SPS genes in Citrus and *Arabidopsis*. Our findings revealed the distribution of *CsSPSs* on three chromosomes: *CsSPS1* and *CsSPS2* on chromosome 1, *CsSPS4* on chromosome 9, and *CsSPS3* on an unidentified chromosome (**Figure 3B**). Synteny analysis between *SPS* genes in citrus and *Arabidopsis* identified *AtSPS1F-CsSPS1* and *AtSPS2F-CsSPS2* as syntenic gene pairs (**Figure 3C**).

### Protein motif, conserved domain, gene sequence, and promoter analysis of *CsSPSs*

**Figure 4:**
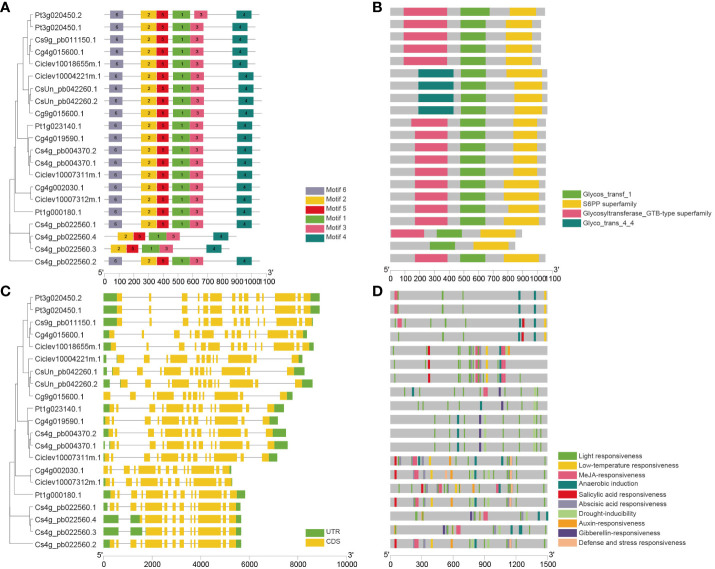
Conserved motifs, domains, gene structure, and predicted cis-elements of *CsSPSs*. **(A)** Conserved motifs (numbers 1–6) are highlighted using different coloured boxes. **(B)** Conserved protein domain of CsSPSs. **(C)** Exon–intron structure of *CsSPSs*. Green boxes (5ʹ UTR and 3ʹ UTR), yellow boxes (exons), and grey lines (introns). **(D)** Predicted cis-elements in *CsSPSs* promoters are represented using different colours.

Furthermore, an analysis of conserved motifs and domains was conducted using the MEME web server, revealing a total of six conserved motifs and three conserved domains in citrus SPS proteins (**Figure 4A, B**). The results indicate that all citrus SPS proteins share the same motifs (**Supplementary Table S3**) and domains: a Glycos_transf domain, an S6PP domain (C-domain), and a Glycosyltransferase domain (N-domain). Notably, citrus SPS3 possesses a Glycos_transf_4_4 domain that other SPS proteins lack. Concurrently, two transcripts of *CsSPS2* (Cs4g_pb022560) lack Motif 6, and one transcript even lacks the Glycos_transf domain, suggesting that these two transcription modes may not be the primary transcription mode of *CsSPS2*.

The exon–intron structures of Cs*SPSs* were further analysed. Generally, most citrus *SPS* genes contain twelve to fourteen exons (**Figure 4C**). Each citrus *SPS* within the same group shares the same intron–exon structure. For instance, all citrus *SPS4* genes contain 14 introns, and every citrus *SPS3* gene contains 12 introns. Additionally, each of *SPS1* and *SPS2* contains 13 introns, except for the two specific transcription modes of *CsSPS2*.

To gain further insights into the functions of *CsSPSs*, the 1500 bp promoter regions upstream of the *CsSPSs* initiation transcription site were analysed using the PlantCARE website (http://bioinformatics.psb.ugent.be/webtools/plantcare/) (**Figure 4D**). The analysis revealed that cis-elements in the promoter region of all identified *CsSPSs* are primarily involved in responding to light, hormones, and abiotic stress. All *CsSPS* promoter regions feature photoresponsive elements (Box 4 and G-box), and most *CsSPS* promoters contain low-temperature, methyl jasmonate (MeJA), and salicylic acid (SA) responsive elements, enabling them to respond to ethylene and abscisic acid (ABA) reactions (**Supplementary Table S4**). The diversity of cis-acting elements in the upstream promoter region of *CsSPSs* indicates that their function may involve various reactions, such as hormones, abiotic stress, seed, and endosperm development.

### Expression profiles of *CsSPSs* in citrus

**Figure 5:**
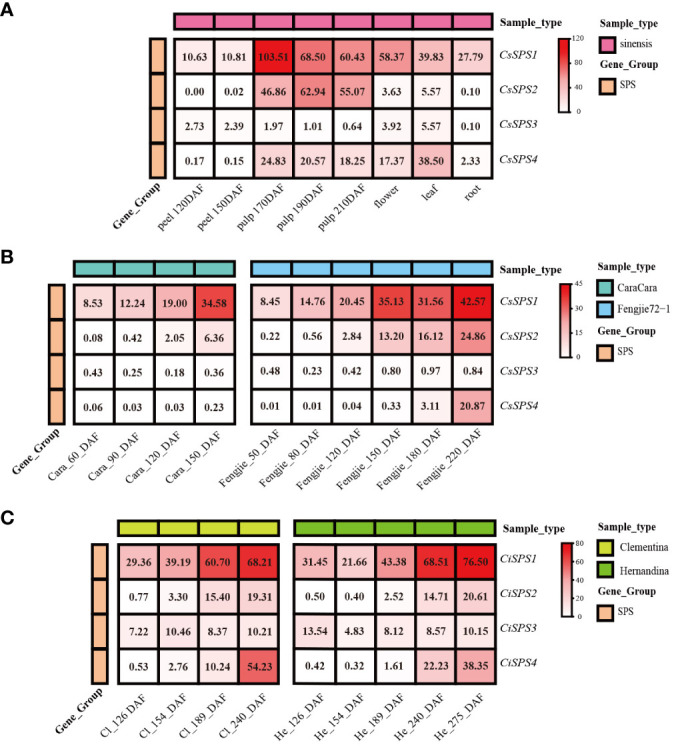
Expression of *CsSPSs* in citrus tissues and varieties. **(A)** Transcription levels in peel, pulp, flower, leaf, and root of ‘*sinensis*’. **(B)** Transcription levels in the pulp of ‘CaraCara’ and ‘Fengjie 72-1’. **(C)** Transcription levels in Clementina and ‘Hernandina’.

Publicly available transcriptomic data revealed distinct expression patterns of *CsSPSs* in different citrus tissues. *CsSPS1* and *CsSPS2* were predominantly expressed in ‘*sinensis*’ pulp, with *CsSPS1* decreasing and *CsSPS2* increasing during fruit maturation (**Figure 5A**). *CsSPS3* and *CsSPS4* exhibited high expression in leaves, with *CsSPS3* also detected in flowers. However, transcriptional expression of *CsSPSs* in peel and root was low. Across maturing citrus fruits, *CsSPSs* displayed unique expression characteristics (**Figures 5B, C**). For example, the transcription level of *CsSPSs* in ‘Fengjie72-1’ is significantly higher than that in ‘CaraCara’, whereas the transcription level of *CsSPSs* in Clementina and Hemandina is similar. The transcriptional expression levels of most *CsSPSs* in pulp were continuously increased. *CsSPS1* and *CsSPS2* showed an evident upward trend in the four varieties. However, some *CsSPSs*, such as *CsSPS3* in ‘CaraCara’, have decreased expression levels, which reflects the tissue expression specificity of *CsSPSs* mentioned earlier.

### Correlation analysis between sugar and *CsSPSs* in citrus fruit

**Figure 6:**
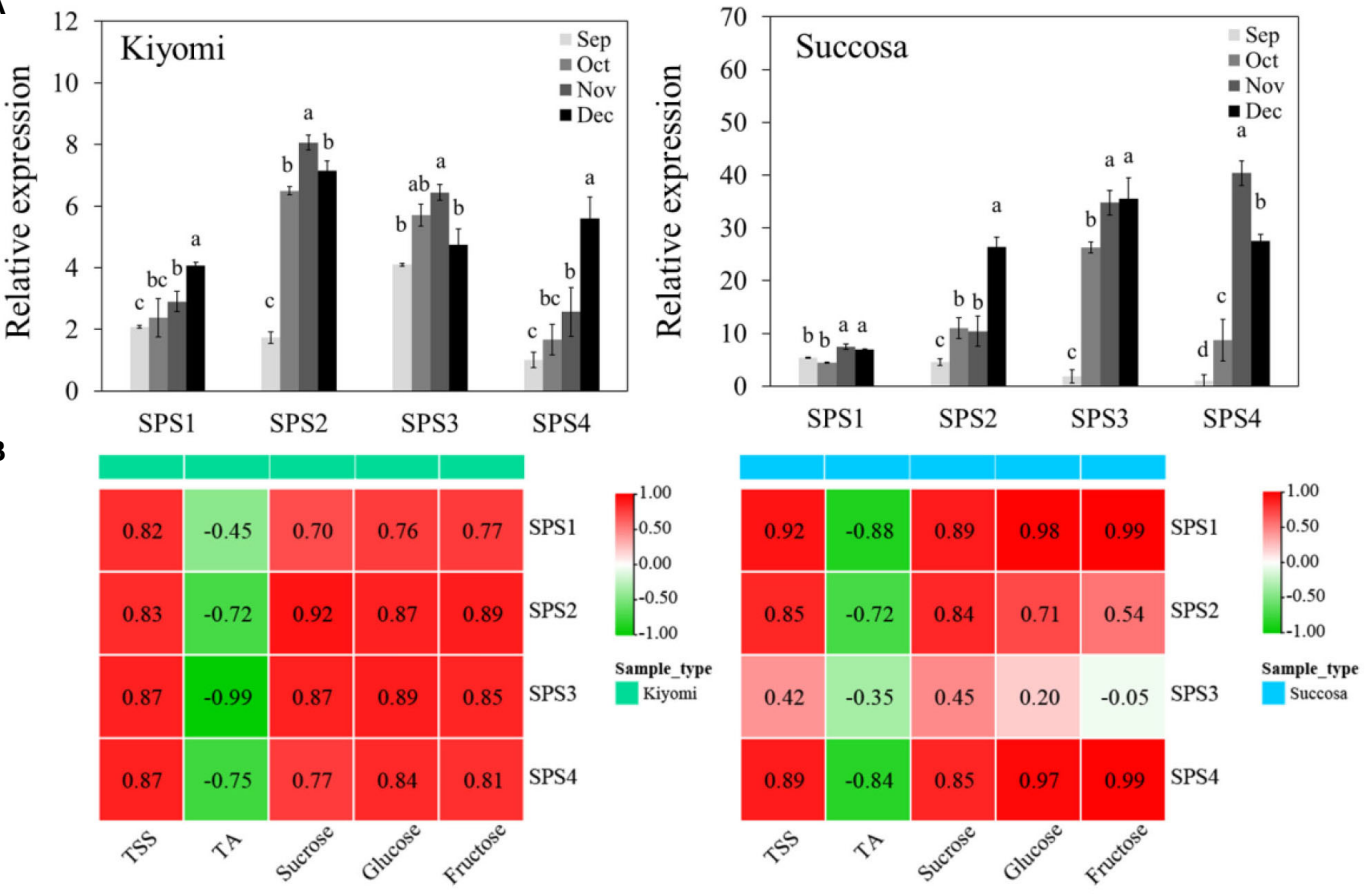
Expression of *CsSPSs* in ‘Kiyomi’ and ‘Succosa’. **(A)** Expression levels. **(B)** Correlation analysis with TSS, TA, sucrose, glucose, and fructose.

To understand the expression of *CsSPSs* in citrus pulp, we investigated the transcriptional expression of *CsSPSs* in ‘Kiyomi’ and ‘Succosa’ (**Figure 6A**). Consistent with the transcriptome results, the transcriptional expression of the four *CsSPSs* in citrus pulp increased with fruit development, and the types of *CsSPSs* expression patterns differed in the pulp of different citrus varieties. For example, The transcription expression of *CsSPS2* and *CsSPS3* is dominant in ‘Kiyomi’, while the transcription expression of *CsSPS3* is dominant in ‘Succosa’. Unexpectedly, *CsSPS1* is considered to be the most important SPS gene in the pulp of other citrus varieties, but in the pulp development of ‘Kiyomi’ and ‘Succosa’, the relative transcriptional expression of *CsSPS1* is lower than that of other *CsSPSs*. Simultaneously, the stable, high transcription level of *CsSPS3* and the rapid increase in *CsSPS4* expression levels in the two materials indicated the importance of *CsSPS3* and *CsSPS4* in the development of citrus pulp.

To comprehensively highlight the significance of *CsSPSs* in citrus sugar accumulation, we analysed Pearson’s correlation coefficients between *CsSPSs* expression and sugar content (**Figure 6B**). As expected, most *CsSPSs* were significantly and positively correlated with sugar content and its components. However, in ‘Succosa’, *CsSPS1* with low transcriptional expression level and *CsSPS4* with late rapid elevation demonstrated the most significant positive correlation with sugar content. Counterintuitively, despite having the highest transcriptional expression level, *CsSPS3* had a low correlation with sugar content.

### Co-expression network analysis of candidate *CsSPSs* during fruit development

**Figure 7:**
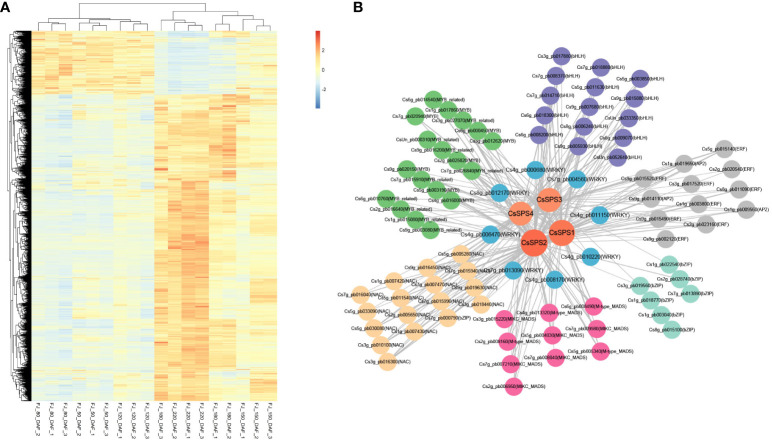
Weighted gene co-expression network analysis of *CsSPSs*. **(A)** Expression of genes in the blue module. **(B)** Co-expression network analysis of *CsSPSs*. (*CsSPSs*. Orange circles, *WRKYs*. Blue circles, *MYBs*. Green circles, *bHLHs*. Purple circles, *ERFs*. Grey circles, *bZIPs*. Turquoise circles, *MADS*. Pink circles, *NACs*. Yellow circles).

To further understand the possible regulatory pathways of citrus sugar metabolism, we analysed the co-expression of *CsSPSs* based on our previous study (Zhang et al., 2023a). The results showed that all four *CsSPSs* were in the blue module (4,871 genes), which was positively correlated with the total sugar content, and the expression levels of most genes in the blue module increased with fruit development (**Figure 7A**). Importantly, in the blue module, we observed 196 transcription factors (**Supplementary Table S6**) that have a significant co-expression relationship with *CsSPSs* (weight > 0.15), including *bHLH* (15), *ERF* (12), *bZIP* (7), *MADS* (10), *NAC* (17) and *MYB* (18) (**Figure 7B**). It is worth noting that we found that eight *WRKY* transcription factors (*CsWRKY20*, *CsWRKY47*, *CsWRKY32*, *CsWRKY2*, *CsWRKY65*, *CsWRKY3*, *CsWRKY28*, *CsWRKY74*) have a co-expression relationship with *CsSPSs*. A positive relationship between *CsWRKY47*, *CsWRKY3*, *CsWRKY28*, *CsWRKY74* and sugar was found in our previous study, indicating that there may be a regulatory relationship between *CsWRKYs* and *CsSPSs.*

### Expression analysis of *CsWRKYs* and dual-luciferase activity assay

**Figure 8:**
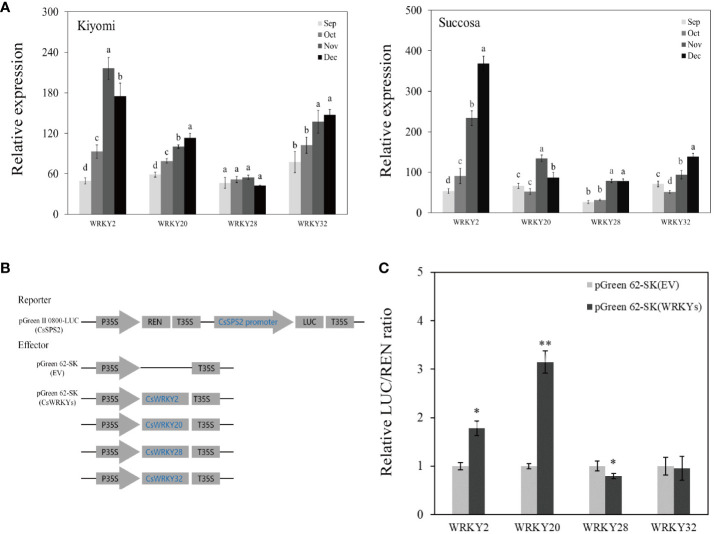
Expression analysis of *CsWRKYs*, Dual-luciferase activity assay of *CsWRKYs* and *CsSPS2* promoter. **(A)** Expression of *CsWRKYs* in ‘Kiyomi’ and ‘Succosa’. **(B)** Schematic diagrams of vectors used for the dual-luciferase activity assay. **(C)** The relative LUC/REN ratio of pGreen II 0800-LUC (*CsSPS2*) × pGreen 62-SK (EV, empty vector) as the control and pGreen II 0800-LUC (*CsSPS2*) ×pGreen 62-SK(*CsWRKYs)*. Asterisks indicate significant differences as determined by Student–Newman–Keuls multiple range test at P < 0.01 level.

To elaborate on the potential involvement of *CsWRKYs* in the sugar metabolism of citrus fruits through *CsSPSs*, we examined the expression of co-expressed *WRKY* genes in Kiyomi’ and ‘Succosa’. The results revealed a continuous increase in the transcriptional expressions of *CsWRKY2*, *CsWRKY20*, *CsWRKY28*, and *CsWRKY32*, and *CsWRKY47* with the maturation of citrus pulp (**Figure 8A**).

Notably, W-box cis-elements was found in *CsSPS2* promoter, but not in other *CsSPSs* (**Supplementary Table S4**). The dual-LUC activity assay between *CsWRKYs* and *CsSPS2* indicated that the co-expression of *CsWRKY2*, *CsWRKY20* with the *CsSPS2* promoter significantly enhanced the LUC to REN ratio, and co-expression of *CsWRKY28* with the *CsSPS2* promoter significantly reduced the LUC to REN ratio, whereas no significant LUC to REN ratios were observed in the independent co-expression of *CsWRKY32* with the promoters of *CsSPS2*. This indicated that *CsWRKY2*, *CsWRKY20* might act as an activator in citrus fruit of sugar accumulation through *CsSPS2*, while *CsWRKY28* inhibits sugar accumulation through *CsSPS2* (**Figure 8C**).

## Discussion

Similar to most citrus fruits, the sugar accumulation in citrus fruits (‘Kiyomi’ and ‘Succosa’) increases during fruit development, with sucrose being the predominant soluble sugar component (Ren et al., 2023). However, distinct citrus varieties exhibit varying rates of sucrose accumulation (**Figure 1B**). The rapid increase in glucose and fructose content, particularly in ‘Kiyomi’, contributes to the swift sucrose accumulation, whereas the stable substrate content in ‘Succosa’ leads to a slower rate of sucrose accumulation (**Figure 2**).

*SPS* genes identified in various plant species, and investigated in this study, revealing four *SPS* genes in representative citrus species (*Citrus sinensis, Citrus clementina, Citrus grandis, Pitrus trifoliat*) (**Figure 3A**). *CsSPS1* and *CsSPS2*, belonging to subfamily A, exhibit high homology with *Arabidopsis AtSPS1* and *AtSPS2* (**Figure 3C**). *CsSPS3* and *CsSPS4* belong to subfamilies B and C, respectively. All *CsSPSs* share typical SPS family domains and similar intron–exon structures, indicating functional similarity among citrus *SPSs* (**Figures 4A-C**). The promoters of *CsSPSs* exhibit responsiveness to light, hormones, and abiotic stress, suggesting their pivotal role in citrus sugar signal responses to external abiotic cues (**Figure 4D**).

As a rate-limiting enzyme in sucrose metabolism, the transcriptional expression of *SPS* directly influences sucrose synthesis (Ruan, 2014; Wan et al., 2018). *CsSPSs*, akin to other species, generally exhibit increased transcriptional expression levels with fruit ripening, showing a significant positive correlation with fruit sugar content (**Figure 6**). However, *CsSPSs* display expression specificity in different tissues and varieties. For example, *CsSPS1* and *CsSPS2* are predominantly expressed in citrus fruits, whereas *CsSPS3* and *CsSPS4* are expressed in leaves and flowers (**Figure 5**). However, *CsSPS3* is the dominant *CsSPS* expression type in ‘Kiyomi’ and ‘Succosa’ fruits. In addition, the expression level of *CsSPS2* in ‘Kiyomi’ was significantly higher than ‘Succosa’, which may be one of the reasons why the sucrose content of ‘Kiyomi’ increased faster than ‘Succosa’. Remarkably, the expression levels of *CsSPS4* increase rapidly during late fruit development in several citrus varieties. Therefore, we believe that *CsSPS1* and *CsSPS2* play a major role in sugar accumulation in citrus fruits, while *CsSPS4* plays a vital role in the late development of citrus fruit. To sum up, the expression types and patterns of *CsSPSs* in different citrus varieties and tissues are different.

Sugar signals play an important role in plant development and resistance to external stress (Gill and Tuteja, 2010; Sperdouli and Moustakas, 2012; Proels and Hückelhoven, 2014). But the synthesis of sugar are subject to regulation by various transcription factors. Silencing strawberry *FaGAMYB* and *FaMYB10* has been shown to reduce the transcriptional expression of *FaSPS3* and *FaSPS1*, respectively (Vallarino et al., 2015). *FaMYB44.2* can directly bind to the *FaSPS3* promoter to inhibit sucrose accumulation (Wei et al., 2018). However, WRKY transcription factors can also be involved in the regulation of sugar metabolism. In the present study, we found that Cs*WRKY2*, *CsWRKY20*, *CsWRKY28* and *CsWRKY32* had a significant positively correlated with *CsSPSs* (**Figure 7**). In previous research, *WRKY2* was found to regulate circadian expression, mediated seed germination, regulation of pollen development, and play an important role in regulating tolerance to abiotic stresses such as cold damage and drought (Jiang and Yu, 2009; Niu et al., 2012; Lei et al., 2017; Wang et al., 2022); Rice *OsWRKY47* and soybean *GsWRKY20* mainly respond to drought stress (Luo et al., 2013; Ning et al., 2017; Li et al., 2023); *WRKY28* is thought to be involved in plant salt tolerance, element absorption and morphogenesis (Yang et al., 2022; Zhang et al., 2023b, Zhang et al., 2023c); Tomato *SlWRKY32* can regulate ethylene signal and affect tomato fruit colouring (Zhao et al., 2021); Nonetheless, There is no doubt that the promoter activity of CsSPS2 can be activated by *CsWRKY2* and *CsWRKY2*, restrained by *CsWRKY28*. Therefore, we suggest that *CsWRKY2*, *CsWRKY20* and *CsWRKY28* may affect the sugar quality of citrus fruit by regulating *CsSPS2*.

## Conclusion

In conclusion, we investigated the sugar composition in ‘Kiyomi’ and ‘Succosa’ and elucidated the complexities of sugar quality formation in citrus fruits. Simultaneously, we conducted a comprehensive identification of *SPS* genes in the citrus genome, revealing four *CsSPSs* belonging to three types with conserved sucrose phosphate synthase domains. Our expression analysis demonstrated that most *CsSPSs* exhibit increased expression levels with citrus fruit maturity, tightly correlated with sucrose content. *CsSPSs* manifest specific expression patterns in different tissues and citrus varieties, with *CsSPS4* emerging as a key player in late fruit development. These findings highlight the pivotal role of *CsSPSs* in citrus fruit sugar metabolism. Notably, building upon our previous studies, we suggest that the identified *CsWRKYs*, particularly *CsWRKY2*, *CsWRKY20*, *CsWRKY28* and *CsWRKY32*, may participate in regulating *CsSPS* expression. *CsWRKY20* might act as an activator in citrus fruit of sugar accumulation through *CsSPS2*, and *CsWRKY28* might can inhibits sugar accumulation through *CsSPS2*. Our results not only elucidate the process of sugar quality formation in citrus fruits but also provide novel insights into the mechanisms through which WRKY transcription factors modulate sugar metabolism.

## Data availability statement

The original contributions presented in the study are included in the article/**Supplementary Material**. Further inquiries can be directed to the corresponding authors.

## Author contributions

WL: Investigation, Resources, Formal Analysis, Writing – original draft. WH: Investigation, Resources, Writing – original draft. KL: Investigation, Resources, Writing – original draft. JL: Investigation, Writing – original draft. CY: Investigation, Writing – original draft. YS: Investigation, Writing – original draft. ZH: Investigation, Writing – original draft. BP: Resources, Supervision, Writing – review & editing. HL: Resources, Supervision, Writing – review & editing. BX: Resources, Supervision, Writing – review & editing. LL: Resources, Supervision, Writing – review & editing. JH: Resources, Supervision, Writing – review & editing. MZ: Conceptualization, Funding acquisition, Investigation, Resources, Validation, Writing – review & editing. XW: Conceptualization, Writing – review & editing. ZW: Conceptualization, Writing – review & editing.
